# Pyrazinamide susceptibility testing in *Mycobacterium tuberculosis*: impact of false-positive BD MGIT 960 results and added value of sequencing for accurate diagnosis in a low-MDR-TB-incidence context

**DOI:** 10.3389/fcimb.2026.1777714

**Published:** 2026-05-14

**Authors:** Touyana Semenova, Grégory Gonzalez, Benoît Lechartier, Peter Sander, Christophe von Garnier, Bettina Schulthess, Jesica Mazza-Stalder, Gilbert Greub, Onya Opota

**Affiliations:** 1 Institute of Microbiology, Lausanne University and Lausanne University Hospital, Lausanne, Switzerland; 2 Microbiology Laboratory, Unilabs, Coppet, Switzerland; 3 Division of Pulmonology, Department of Medicine, Lausanne University and Lausanne University Hospital (CHUV), Lausanne, Switzerland; 4 Institute of Medical Microbiology, University of Zurich, Zurich, Switzerland; 5 National Reference Center for Mycobacteria, Zurich, Switzerland; 6 Infectious Disease Service, Lausanne University and Lausanne University Hospital, Lausanne, Switzerland

**Keywords:** Antibiotic resistance, antibiotic susceptibility testing, MDR-TB, molecular diagnosis, *Mycobacterium bovis*, pyrazinamide, Sanger and next-generation sequencing, *Mycobacterium tuberculosis complex*

## Abstract

**Introduction:**

The reliability of phenotypic pyrazinamide (PZA) drug susceptibility test (DST) in *Mycobacterium tuberculosis* complex (MTBC) isolates is under growing scrutiny. The MGIT 960 System, the only WHO-endorsed method, has shown increasing false positive (FP) rates. In 2024, we reported a surge, and in 2025 Becton, Dickinson, and Company (BD) issued a global alert and suspended the PZA kit production. We assessed the extent of FP PZA resistance (PZA-R), its diagnostic impact, and the ways to distinguish FP from true positive (TP) results in our laboratory.

**Results:**

Between 2023 and 2025, 9% of the isolates were confirmed as TP PZA-R, mostly due to *M. bovis*. The MGIT 960 FP rate rose from 18% in 2023 to 33% in early 2025. Among 176 isolates, 47 (27%) were FP, 16 (9%) were TP, and 112 (64%) were true negatives (TN). The TP isolates had a longer TTP than the FP isolates (7.2 vs 5.7 days). The FP isolates showed MGIT intermediate growth index values between TP and TN (*p* < 0.005). Notably, the H37Rv reference strain had a 25% FP rate. The confirmatory testing increased the workload but prevented misreporting.

**Conclusions:**

Our data reveal a high FP rate for PZA-R with the MGIT 960 System, with no reliable phenotypic alternative available. The sequencing of *pncA* has been crucial in preventing misreporting and ensuring accurate TB treatment decisions. There is an urgent need for better phenotypic tools and broader molecular approaches beyond *pncA* so that PZA-R can be detected more reliably and on a scale that supports everyday clinical practice.

## Graphical Abstract

**Figure:**
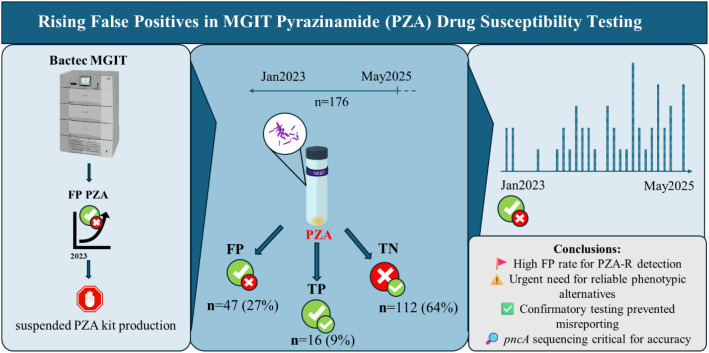

## Introduction

Pyrazinamide (PZA) is a first-line drug active on the *Mycobacterium tuberculosis* complex (MTBC) with dose-dependent bacteriostatic and bactericidal activity. Its unique sterilizing effect helps shorten the treatment duration and enhance efficacy, particularly during the intensive phase (Donald et al., 2012; Chigutsa et al., 2013; Lamont et al., 2020). PZA is a prodrug converted into its active form, pyrazinoic acid (POA), by the enzyme pyrazinamidase (PZase), encoded by *pncA*. Resistance typically arises from *pncA* mutations that impair POA production (McClatchy et al., 1981; Butler and Kilburn, 1983; Wu et al., 2019). However, approximately 30% of phenotypically resistant isolates have wild-type *pncA* and functional PZase, suggesting additional, unknown resistance mechanisms (Zhang et al., 1999; Bhuju et al., 2013; Wu et al., 2019, Shi et al., 2022).

PZA is only active in acidic conditions; thus, culture-based testing drug susceptibility test (DST) for PZA must be performed at a pH level of 5.5 and lower (Zhang et al., 2013; Mok et al., 2021). PZA DST can be performed using phenotypic methods, such as the mycobacteria growth indicator tube (MGIT) 960 System Becton, Dickinson, and Company (BD) and PZase activity, or genotypic methods, which detect mutations in the *pncA* gene and its promoter region (Scorpio and Zhang, 1996; Juréen et al., 2008; Ramirez-Busby and Valafar, 2015; Tam et al., 2019). The only WHO-recommended technique for phenotypic PZA drug DST at the moment is the BACTEC™ MGIT™ 960 system (World Health Organization, 2018). However, several studies have highlighted concerns regarding the accuracy of this assay (Zhang et al., 2002; Morlock et al., 2017; Ghodousi et al., 2025; Lee et al., 2014; Mustazzolu et al., 2017). In 2024, we reported a rise in false-positive (FP) PZA-R to BD. Finally, in 2025, BD issued a global alert regarding an increase in FP results and announced the suspension of the MGIT PZA kit production effective May 2025.

This study aimed to assess the performance and limitations of current phenotypic and genotypic methods for PZA susceptibility testing and to explore alternative approaches for reliable detection. We retrospectively analyzed clinical MTB isolates tested with the MGIT 960 system from January 2023 to May 2025.

## Materials and methods

### *M. tuberculosis* clinical isolates and quality control strains

A total of 176 MTB isolates tested for pyrazinamide susceptibility between January 2023 and May 2025 were retrospectively included. The study population comprised all consecutive MTB isolates from clinical samples as part of the routine diagnostic process at the Institute of Microbiology, Lausanne University Hospital. Clinical samples were received from both inpatient units and outpatient clinics. As a result, the sample size reflected our routine laboratory activity in a low-TB-incidence setting rather than by a predefined sample size. The H37Rv PZA-susceptible strain is used routinely as an internal quality control (IQC) and is globally used as the TB reference sequence (Santoro et al., 2017). In total, 176 isolates were analyzed, including 18 runs of the H37Rv strain (Cole et al., 1998). Additionally, four samples from external quality assessment (EQA) panels provided by the Association for Medical Quality Control Zurich (MQZH) were evaluated.

### BACTEC MGIT 960

Firstly, the clinical specimens were incubated in the BACTEC™ MGIT™ 960 system (Becton, Dickinson, and Company, NJ, United States) until flagged as positive or for a maximum duration of 42 days, after which the cultures with no detectable growth were classified as negative according to the manufacturer’s instructions. Then, all TB-positive isolates were tested for PZA susceptibility using the BACTEC™ MGIT™ 960 PZA kit as part of routine diagnostic workflows following the furnisher’s instructions. Briefly, a strain is considered PZA susceptible if growth in the drug tube was inhibited compared to the control (growth unit value of 400 of a 1:10 diluted inoculum) and resistant if growth in the drug-containing tube reached 100 growth units earlier than when the control reached 400 growth units. PZA time to positivity (TTP) was defined as the duration, in days, from the start of incubation of the MGIT tubes with PZA to the time when the instrument flagged the culture as susceptible or resistant. For the MGIT growth index, we analyzed the data on the obtained growth concentration and compared them among FP, true-positive (TP), and true-negative (TN) samples.

### Sequencing of *pncA* and *gyrB* genes

Confirmatory testing included repeat MGIT assays and targeted Sanger sequencing of *gyrB* genes in our laboratory and the *pncA* at the Institute of Medical Microbiology, University of Zurich (Zurich, Switzerland). The sequencing of *gyrB* enabled the identification of *Mycobacterium bovis*, a member of MTBC with intrinsic resistance to PZA due to a specific *pncA* mutation altering the codon His57Asp. Samples were classified as FP, TP, or TN based on the obtained *pncA* and *gyrB* sequencing results. TP isolates were considered PZA-R when both phenotypic and genotypic methods confirmed the presence of resistance. Isolates were classified as FP in the case when phenotypic testing indicated PZA-R, while *pncA* sequencing did not reveal any resistance-related mutations.

Sequencing of the *gyrB* gene was routinely performed in our laboratory using the following primers: forward MtubF 5′-TCGGACGCGTATGCGATATC-3 and reverse MtubR 5′- ACATACAGTTCGGACTTGCG-3′. The resulting amplicons were subjected to *in silico* enzymatic digestion using Geneious Prime^®^ software (Biomatters Ltd.), which enables species prediction based on restriction fragment patterns. This analysis was specifically used to confirm *M. bovis*, which is known for its intrinsic resistance to PZA.

During the study period, *pncA* gene sequencing was outsourced to the Institute of Medical Microbiology, University of Zurich (Zurich, Switzerland). In June 2025, we implemented the assay in our laboratory. The *pncA* gene was amplified using the primers (forward) pncA-F 5′- GCTGGTCATGTTCGCGATCG-3′ and (reverse) pncA-R 5′-CAGGAGCTGCAAACCAACTCG-3′ using the program//1X(95°C 10’)/33X(96°C 3″ - 60°C 3″ - 68 °C 15″)/1x(72 °C 10″)/8°C//. These primers generated an approximately 665-bp amplicon covering the full 561-bp coding region of the *pncA* gene, reference genome H37Rv NC_000962.3. The same primers were used for Sanger sequencing. The amplicons generated by *pncA* gene sequencing were analyzed using several complementary approaches. First, sequences were aligned with the MTBC H37Rv ATCC reference strain (PZA-susceptible, wild-type *pncA*) to detect any deviation from the susceptible genotype, particularly loss-of-function variants, in the *pncA*-coding region and promoter using the WHO catalogue of mutations associated with drug resistance in MTBC (World Health Organization, 2023). Second, the sequences were compared to a *M. bovis* reference carrying the His57Asp substitution, which is known to confer intrinsic resistance to PZA. Third, a specific search for the synonymous 138A>G mutation at codon 46 was conducted to identify potential *M. canettii* strains also intrinsically resistant to PZA.

The biobank strains used for validation had previously been characterized using a composite reference standard combining phenotypic and molecular approaches. This included pyrazinamide susceptibility testing using the MGIT 960 system, targeted sequencing of the *pncA* gene to detect resistance-associated mutations, and assessment of pyrazinamidase (PZase) activity, which evaluates the enzymatic conversion of pyrazinamide into its active form, pyrazinoic acid. The combined interpretation of these methods was used to establish the reference resistance profile of each strain.

## Results

### Rise of false-positive rates in PZA resistance testing (2023–2025)

**Figure 1:**
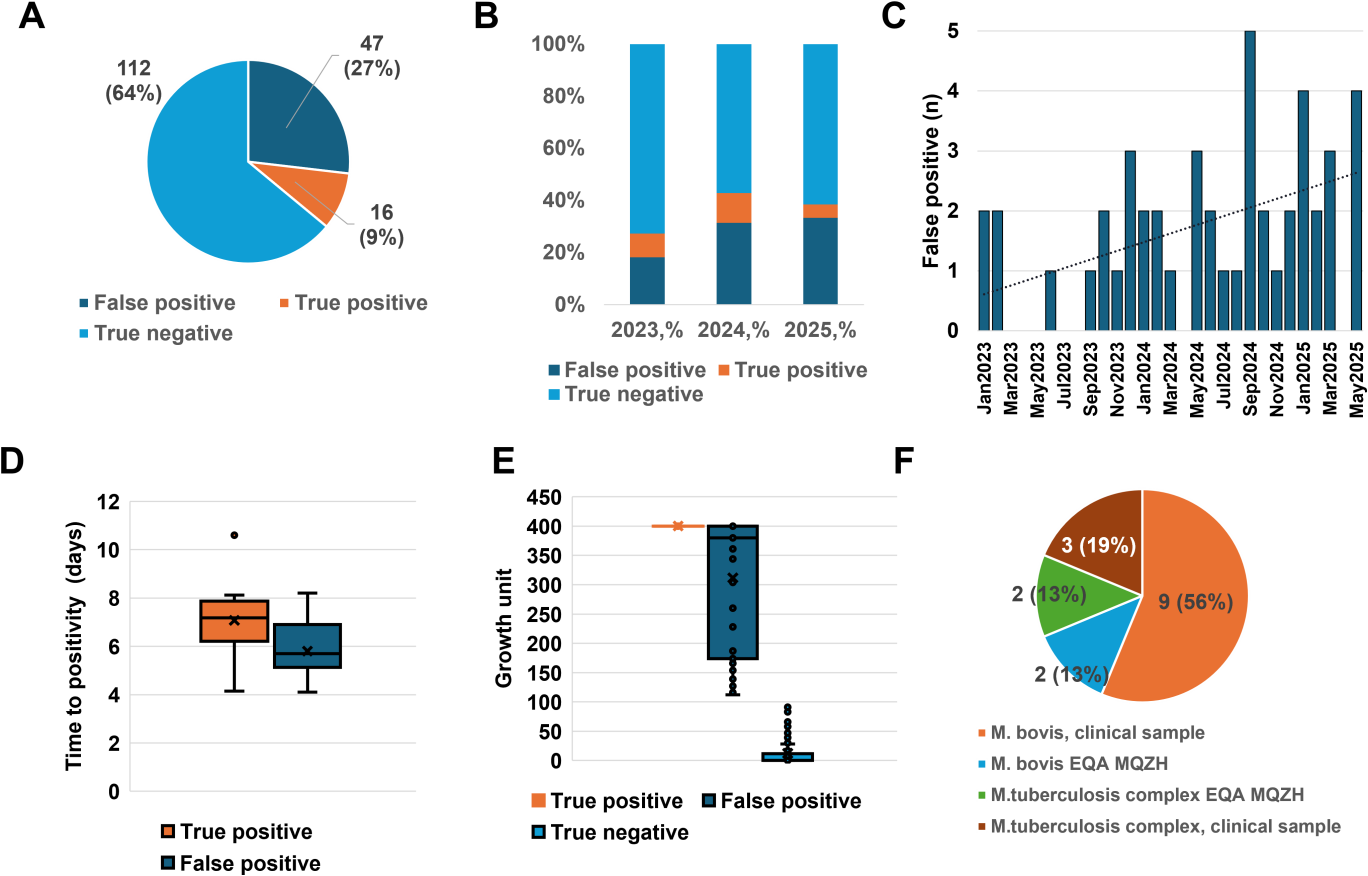
Trends, performance, and characterization of pyrazinamide (PZA). Drug susceptibility testing using the MGIT 960 System (2023–2025). **(A)** Classification of clinical isolates based on phenotypic MGIT DST and confirmed by *pncA* and *gyrB* sequencing (*n* = 176). Among the 176 total isolates, 47 (27%) were FP, 16 (9%) were true positives (TP), and 112 (64%) were true negatives (TN). One isolate (1%) was deemed non-valid due to a mixed culture with *M. avium*, rendering the PZA DST uninterpretable. **(B)** Annual distribution of clinical and quality control isolates based on PZA DST results per year (*n* = 176). The false positive (FP) increased from 18% in 2023 to 31% in 2024, reaching 33% during the first five months of 2025 (January–May). **(C)** Monthly occurrence of false-positive (FP) isolates (*n* = 47). FP isolates were detected almost monthly starting from January 2023, with a mean of 1.62 FP cases per month. **(D)** Comparison of mean time to positivity (TTP) in the MGIT system between TP and FP *Mycobacterium tuberculosis* complex (MTB) isolates. The mean TTP was significantly longer for TP isolates (7.2 days) compared to FP isolates (5.7 days), with the difference reaching statistical significance (*p* = 0.0039) but with an overlap between the two groups. **(E)** Comparison of MGIT growth index among TP, FP, and TN samples. All TP samples exhibited a growth index of 400 units. The FP samples had a lower mean growth index of 311 units, while the TN samples showed minimal growth with a mean of 12 units. The differences between groups were statistically significant (*p* < 0.005). **(F)** Characterization of TP isolates (*n* = 16). The clinical strains included nine (56%) *M. bovis* and three (19%) multidrug-resistant MTB. The external quality assessment (EQA) samples from the Association for Medical Quality Control Zurich (MQZH) included two samples (13%) that were positive for *M. bovis* and two samples (13%) that were positive for MTB.

Between January 2023 and May 2025, among 176 isolates of MTBC tested for PZA using the MGIT DST assay, we reported 16 TP (9%) isolates. However, during the same period, 47 isolates (27%) were initially classified as FP based on discordance between the MGIT DST assay and genotypic results (*pncA*/*gyrB* sequencing), and 112 were classified as TN (64%) (**Figure 1A**). Remarkably, the annual distribution revealed a notable rise in FP rates: from 18% in 2023 to 31% in 2024 and 33% during the first five months of 2025 (**Figure 1B**). FP isolates were detected nearly every month since January 2023, with a monthly mean of 1.62 cases, confirming that this trend is sustained rather than sporadic (**Figure 1C**). These findings suggested a decreasing reliability of phenotypic MGIT-based PZA-R detection, highlighting the need for closer evaluation of DST quality and systematic integration of genotypic confirmation.

### Comparison of phenotypic versus genetic indicators of true- versus false-positive PZA resistance

We further analyzed phenotypic growth characteristics using the MGIT system to try to distinguish true from FP PZA-R (**Figures 1D–F**). The TTP in MGIT was significantly longer for TP isolates (mean, 7.2 days) compared to FP (5.7 days), though with partial overlap (*p* = 0.0039) (**Figure 1D**). We also analyzed the MGIT growth index data for all isolates. The TP samples consistently showed a maximal growth index at 400 units in the MGIT containing 100 µg of PZA. It indicates robust growth despite the presence of the drug. In contrast, the FP samples had a mean growth index of 311, and the TN isolates showed minimal growth, with a mean index of 12. These differences in growth were statistically significant but cannot be used for discrimination because of a strong overlap between the TP and FP isolates (*p* < 0.005) (**Figure 1E**).

Among the 16 confirmed PZA-resistant isolates, 12 originated from routine diagnostic workflows, and four originated from external quality assessment panels. Among the 16 TP isolates, nine (56%) were clinical strains identified as *M. bovis* using *gyrB* sequencing, which is intrinsically resistant to PZA due to a mutation in the *pncA* gene. Two isolates (12.5%) were multi-drug-resistant (MDR) MTBC strains for which PZA-R was also due to a mutation in the *pncA* gene. Only one clinical isolate of MTBC with PZA-R was not identified as *M. bovis*; however, the resistance was associated to a loss-of-function premature stop mutation within the *pncA* gene. Additionally, four TP isolates were EQA strains provided by the MQZH: two isolates were identified as *M. bovis* strains and two as MDR MTBC (**Figure 1F**). In detail, among the 16 isolates confirmed as true pyrazinamide resistant, the majority corresponded to *Mycobacterium bovis* or *M. bovis* BCG carrying the canonical pncA His57Asp substitution, which confers intrinsic resistance to pyrazinamide. In total, 13 isolates belonged to this group, including nine clinical *M. bovis* isolates and two *M. bovis* BCG isolates as well as two strains originating from external quality assessment panels. One *Mycobacterium tuberculosis complex* isolate (non-*bovis*) harbored a loss-of-function nonsense mutation in pncA (Tyr103Stop). In addition, two multidrug-resistant *M. tuberculosis* isolates carried mutations in the *pncA* gene, including one frameshift mutation caused by a nucleotide insertion at position 193 and one missense mutation resulting in a Gly132Ala substitution. These findings are consistent with the known role of *pncA* alterations as the primary mechanism of pyrazinamide resistance in *Mycobacterium tuberculosis complex*.

These findings support the added value of genotypic reassessment to prevent misclassification of resistance. They also highlight that mutation in *pncA* is the primary mechanism of resistance to PZA, which prompted us to implement *pncA* sequencing as part of our routine diagnostic workflow.

### Rapid implementation of *pncA* sequencing as part of our routine diagnostic workflow

**Figure 2:**
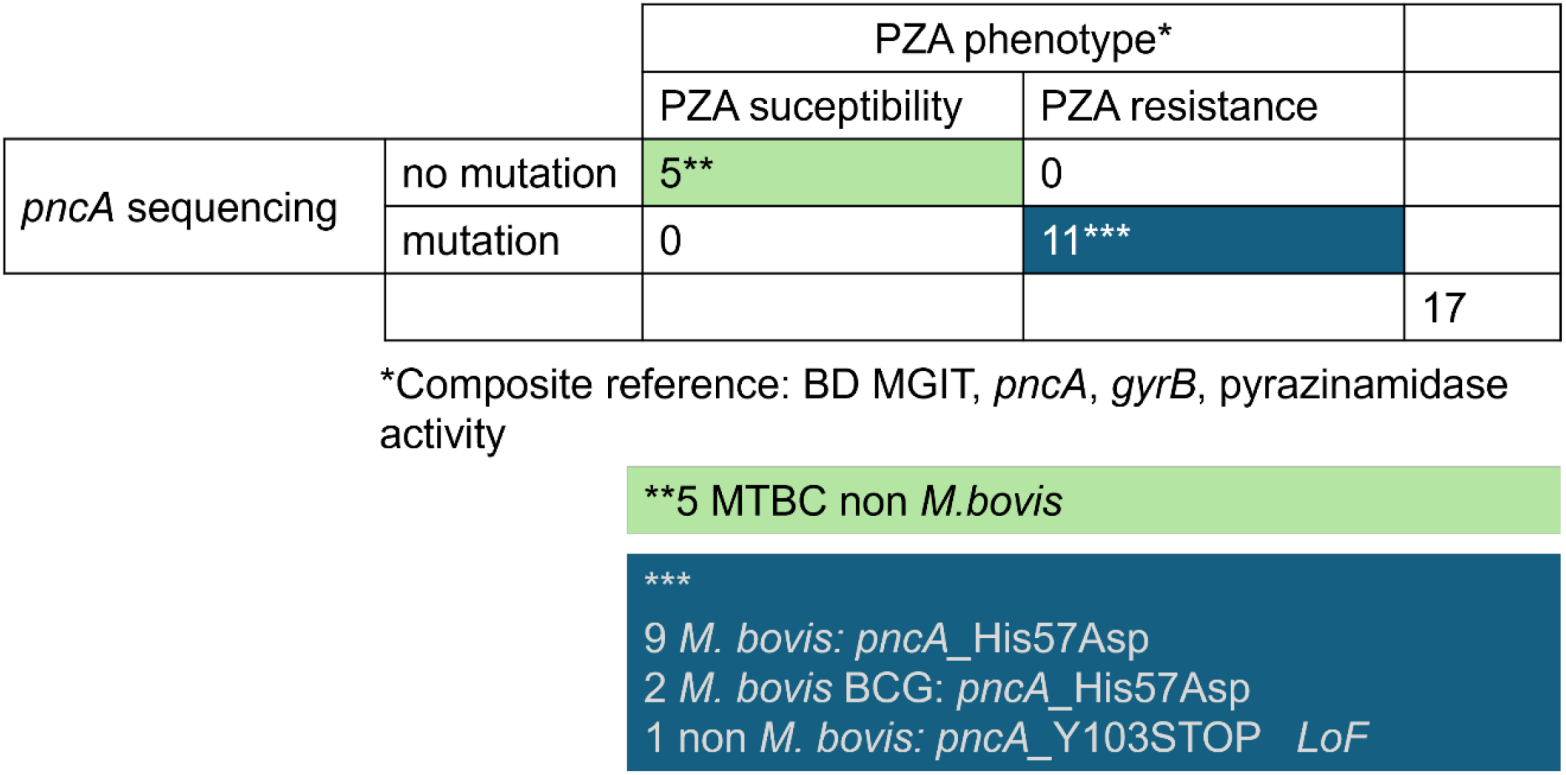
Validation of the implementation of *pncA* sequencing in our laboratory. Validation of *pncA* sequencing using 17 biobank strains with previously characterized pyrazinamide resistance profiles. The reference resistance status had been established using a composite reference standard combining MGIT 960 phenotypic drug susceptibility testing, *pncA* sequencing, and pyrazinamidase activity testing. Among these strains, 11 corresponded to *Mycobacterium bovis* or *M. bovis* BCG carrying the intrinsic resistance mutation *pncA* His57Asp (nine *M. bovis* clinical isolates and two *M. bovis* BCG strains). The remaining isolates belonged to the *Mycobacterium tuberculosis* complex (MTBC) but were not identified as *M. bovis*, including one isolate carrying a loss-of-function mutation (*pncA* Tyr103Stop). The annotations ** and *** indicate the grouping of MTBC non-*bovis* isolates and *M. bovis*-related strains, respectively, in the schematic representation.

To validate *pncA* gene sequencing for routine use in our laboratory, we retrospectively analyzed 17 strains from our biobank. These strains were previously tested for PZA resistance using a composite reference standard that combined phenotypic and molecular methods previously analyzed for *pncA* at the Institute of Medical Microbiology, University of Zurich (Zurich, Switzerland). We observed 100% concordance between the *pncA* sequencing results and the expected resistance profiles, confirming the reliability of the assay (**Figure 2**). Notably, *pncA* sequencing identified a mixed nucleotide signal in one isolate, suggesting the presence of a mixed bacterial population. This finding highlights the importance of careful chromatogram inspection, as mixed populations could compromise PZA efficacy and may otherwise go undetected with standard DST methods.

## Discussion

Our findings raise major concerns about the reliability of phenotypic PZA DST with the MGIT 960 system, as the FP rates rose from 18% to 33% between 2023 and early 2025. These obtained data are particularly relevant with the global alert issued by BD and the subsequent suspension of the MGIT PZA kit production in 2025. As the MGIT 960 System has been the only WHO-recommended phenotypic method for PZA DST, this suspension leaves laboratories without a validated phenotypic alternative. In addition, the high FP rate observed in H37Rv (25% of test runs) highlights the intrinsic limitations of the assay’s robustness and reproducibility. H37Rv is a well-characterized, PZA-susceptible reference strain and used routinely as internal quality control. H37Rv is expected to demonstrate highly reproducible results. Several factors may contribute to the unexpectedly high false-positive rate observed in the MGIT 960 PZA susceptibility assay. Variations in inoculum density may affect bacterial growth dynamics and influence the interpretation of resistance in the system. In addition, variability in medium acidification, which is essential for pyrazinamide activity, may alter drug efficacy and contribute to inconsistent results. Strain heterogeneity or the presence of mixed bacterial populations could also affect phenotypic testing outcomes. Finally, instrument threshold interpretation and the algorithm used for automated growth detection in the MGIT system may influence resistance classification, particularly in cases with borderline growth patterns. Thus, the occurrence of this FP PZA resistance suggests that the variability is related to the assay and not to strain-specific resistance mechanisms. This finding reinforces the need for cautious interpretation of phenotypic PZA DST results and encourages the implementation of genotypic methods into routine diagnostic workflow to avoid any misclassification.

For TB laboratories, this situation has immediate negative consequences. Relying only on MGIT-based DST can overestimate PZA resistance, leading to unnecessary changes in treatment strategy, including PZA removal from the regimen which would otherwise be effective. Given that PZA is essential for shortening treatment duration and possesses a unique sterilizing activity, these diagnostic inaccuracies will have a negative impact on patients’ outcome.

In response to this challenge, we compared diagnostic tools that are used to detect PZA-R and found a 27% FP rate by MGIT despite wild-type *pncA*, highlighting known issues with pH and inoculum sensitivity (Zhang et al., 2002; Chedore et al., 2010; Piersimoni et al., 2013; Morlock et al., 2017; Mok et al., 2021). The FP isolates showed a shorter TTP value (5.7 vs. 7.2 days; *p* = 0.0039), but overlap limits its utility. The growth index also differed (TP: 400 µg/mL; FP: 311 µg/mL; TN: 12 µg/mL), yet 47% of the FP isolates still reached 400 µg/mL, limiting its diagnostic reliability.

In this context, genotypic approaches represent the most reliable alternative. To prevent FP reporting, we used duplicate/triplicate phenotypic testing with *gyrB* and *pncA* sequencing for all PZA-R strains. This improved accuracy but increased workload and delayed results. *gyrB* sequencing identified *M. bovis* in 56% of TP isolates (intrinsically resistant), while two TP isolates were MDR *M. tuberculosis*, requiring further genotypic analysis. These data show that *pncA* sequencing has excellent concordance with reference classifications and allows accurate discrimination between TP and FP phenotypic results. Our findings support the integration of *pncA* sequencing into routine diagnostic workflows as a pragmatic response to the current lack of phenotypic DST options. While the majority of PZA-R is associated with mutations in *pncA*, other genes can also be involved: up to 30% of PZA-R strains represent other than *pncA*-dependent resistance mechanisms: notably, mutations in *rpsA*, *panD*, and *clpC1* as well as putative efflux pumps like *Rv0191*, *Rv3756c*, *Rv3008*, and *Rv1667c* (Carter et al., 2024). Consequently, some FP isolates could, in fact, have alternative resistance determinants—for example, *panD*, which encodes aspartate decarboxylase, is implicated in β-alanine biosynthesis and has also been identified as a target of PZA. Mutations in *panD* have been found in MTB in the absence of *pncA* mutations. These findings suggest an alternative, albeit less common, resistance pathway (Zhang et al., 2013; Shi et al., 2022), although these mechanisms are less frequent and their potential presence underscores the limitations of relying on *pncA* and *gyrB* sequencing. Thus, future studies should include additional resistance-associated genes which could provide a more complete understanding of PZA resistance.

**Table 1** summarizes available methods for PZA susceptibility testing. The MGIT 960 System, once a gold standard, shows poor reproducibility and high FP rates. PZase activity testing offers direct enzymatic assessment but lacks reliability (Cui et al., 2013). *pncA* sequencing offers high sensitivity, specificity, and fast turnaround, but interpretation can be difficult with novel mutations (Miotto et al., 2014; Li et al., 2021). *gyrB* sequencing helps identify *M. bovis* but is limited for resistance detection. NGS provides comprehensive profiling but is costly and complex (Sheen et al., 2017). Systems like Sensititre™ lack PZA, limiting their utility (Lee et al., 2014).

**Table 1:** Available methods for the susceptibility testing of *Mycobacterium tuberculosis* isolates.

Method	Description	Pros	Cons	References
MGIT (mycobacterial growth indicator tube)	Phenotypic method of measuring growth in the presence of PZA	The only method approved by WHO	Requires specialized equipment False-positive resistance due to acidic conditions, inoculum size Labor-intensive	Cui et al., 2013
PZase (pyrazinamidase) activity test	Measures the enzyme activity responsible for PZA activation	Direct functional test Can confirm resistance due to loss of enzyme activity	Variable sensitivity Requires cultured isolates	Cui et al., 2013
*pncA* gene sequencing	Detects mutations in the *pncA* gene associated with resistance	High sensitivity and specificity Rapid turnaround Detects diverse mutations Can detect mixed populations of susceptible and resistant strains	Some mutations have unclear significance Requires sequencing infrastructure	Miotto et al., 2014 Li et al., 2021
*gyrB* gene sequencing	Genotyping of MTBC	Identification of *Mycobacterium bovis*, intrinsically resistant to PZA	Limited direct relevance of PZA-R	
NGS (next-generation sequencing): Amplicon-based targeted NGS WGS (whole-genome sequencing)	Comprehensive sequencing of resistance-associated genes	Detects known and novel mutations Can detect mixed populations of susceptible and resistant strains Can assess multiple drugs at once	High cost Requires bioinformatics expertise Longer turnaround than targeted methods	Sheen et al., 2017
Sensititre System (Thermo Fisher)	Commercial phenotypic MIC-based assay platform for drug susceptibility	Standardized and automated Quantitative MIC results Suitable for multiple drugs	Requires specialized equipment Costly reagents Pyrazinamide is not included	Lee et al., 2014

Recent advances in molecular prediction methods have improved the ability to predict PZA resistance—for example, machine learning models using the structural and sequence features of *pncA* mutations demonstrated better prediction in comparison to simple mutation catalogues (Carter et al., 2024). In addition, large-scale whole-genome sequencing combined with a machine learning model can identify resistance across the genome, including *panD* in isolates with wild-type *pncA* (Pruthi et al., 2024). This demonstrates the potential of modern molecular and computational approaches to complement phenotypic testing and improve diagnostic accuracy. Our study has sample size limitations which restrict the statistical power for subgroup analysis and limits the precision of some additional comparisons. However, this size reflects routine laboratory activity in Switzerland as a low-incidence-TB country where true PZA resistance is uncommon outside *M. bovis* and MDR MTBC. After all, the aim of this study was to evaluate the performance and reliability of phenotypic PZA DST in real-life laboratory practice rather than to estimate resistance prevalence.

The study was conducted in a low-incidence context, reflecting the epidemiological situation of our setting and allowing the assessment of diagnostic performance in conditions where the pre-test probability of resistance is low. Therefore, the findings may not be directly generalizable to settings with higher levels of resistance, which may influence diagnostic test performance. We understand that the obtained results may not be appliable to all epidemiological settings. However, the consistently high FP rate observed across the study period nonetheless highlights important diagnostic challenges with limitations of phenotypic PZA testing that can impact diagnostic routines across diverse epidemiological contexts.

In summary, our data highlight the major limitations of phenotypic PZA DST using the MGIT 960 System. High FP rates, poor IQC reliability, and assay complexity undermine its diagnostic value. With no validated phenotypic alternative, genotypic methods are essential to prevent misclassification and guide the proper use of PZA. Given the limitations of phenotypic methods, *pncA* gene analysis is the best alternative, offering accuracy, speed, and diagnostic value, especially when used with curated mutation databases. Integrating *pncA* sequencing into workflows can ensure reliable PZA-R detection post-MGIT era. Taken together, these findings reflect real-world evidence and highlight the urgent need for revised diagnostic strategies for PZA susceptibility testing.

## Data Availability

The raw data supporting the conclusions of this article will be made available by the authors upon request, in accordance with ethical considerations.
